# Increasing the Fatigue Resistance of Strain-Hardening Cement-Based Composites (SHCC) by Experimental-Virtual Multi-Scale Material Design

**DOI:** 10.3390/ma14195634

**Published:** 2021-09-28

**Authors:** Dominik Junger, Johannes Storm, Steffen Müller, Michael Kaliske, Viktor Mechtcherine

**Affiliations:** 1 Institute of Construction Materials, Technische Universität Dresden, 01187 Dresden, Germany; steffen.mueller@tu-dresden.de (S.M.); viktor.mechtcherine@tu-dresden.de (V.M.); 2 Institute of Structural Analysis, Technische Universität Dresden, 01187 Dresden, Germany; johannes.storm@tu-dresden.de (J.S.); michael.kaliske@tu-dresden.de (M.K.)

**Keywords:** SHCC, ECC, strain-hardening, cyclic loading, numerical modelling, fatigue

## Abstract

Strain-hardening cement-based composites are a promising class of materials for a wide variety of applications due to their considerable tensile strength and pronounced ductility caused by the development of multiple fine cracks. Nevertheless, the safe use of such composites requires sound knowledge of their mechanical behaviour under different types of loading, particularly under fatigue loading, while considering distinct influences like initial crack width and fibre orientation. To deepen this knowledge, single-fibre pull-out tests on PVA-fibres from a cementitious matrix were carried out to gain information about the micro-mechanical and degradation processes of the fibre. It could be shown that the fibres tend to rupture instead of being pulled out under quasi-static loading. When changing the loading regime to alternating loading, this failure mechanism shifts to pull-out. By varying the experimental parameters such as initial crack width, inclination angle or compressive-force level a clear influence on the fibre’s crack bridging capacity could be observed associated with effects on the degradation processes. Based on the data obtained, a micro-mechanical numerical model was developed to support the assumptions and observations from single-fibre pull-out tests and to enable predictions of the performance of the material on the microscale under cyclic loading.

## 1. Introduction

Strain-hardening cement-based composites (SHCC), also known as engineered cement-based composites (ECC), are a class of fibre-reinforced construction materials tailored by micro-mechanical design [1] to achieve increased tensile strength and remarkably high strain capacities of several percent [2], the latter due to multiple cracking behaviour with crack widths below 100 µm [3]. Such pronounced inelastic deformations also give rise to high energy absorption capacity [4]. The development of multiple cracks is the result of the transfer of stresses via the cracks by the fibres associated with stress redistributions and the development and growth of new cracks [5]. Hence, strain-hardening cement-based composites are favourable for applications in dynamic loading regimes. In particular, the material response under high strain rates, e.g., under impact loads caused by collisions or explosions, is a prominent topic of recent research [4,6,7,8,9]. Furthermore, seismic activities represent a serious risk for structures in earthquake-prone areas since the high amplitude vibrations damage structures permanently. In such situations the application of SHCC provides a higher level of safety [10,11]. It could also be shown that the composite represents a good repairing material for existing construction due to its promising load-bearing capacity and the small crack widths due to the high ductility [12,13].

In addition to the loading scenarios described, sound knowledge of the long-term cyclic behaviour of strain-hardening cement-based composites is required for their secure use in construction, since many structures are exposed to repetitive loads. Müller and Mechtcherine [14,15,16] and Jun and Mechtcherine [17] investigated the cyclic behaviour of PVA–SHCC and revealed several degradation mechanisms caused by the cyclic load, mainly at the macroscopic level. Beside investigations on the cyclic response of plain SHCC, several researchers also investigated the behaviour of SHCC reinforced with steel or FRP rebars under alternating loading regimes [18,19,20,21]. For a better understanding of the phenomena observed, micromechanical tests must be performed. The article at hand is intended to summarize the micromechanical experimental investigations by Ranjbarian and Mechtcherine [22,23,24,25] and Ranjbarian et al. [26,27,28]. Based on the experimental data, a numerical simulation tool was developed to enable the validation of experimental assumptions and observations and, finally, facilitate prediction of the performance of SHCC and optimisation of the composite material.

The performed experiments and observed phenomena on cyclically loaded PVA fibres embedded in a cementitious matrix represent an important basis for the comprehensive understanding of strain-hardening cement-based composites under fatigue loads. In conjunction with the developed numerical model the work contributes to the future application of the material in the construction industry.

## 2. Materials and Methods

### 2.1. Materials

For the investigations summarised in this article, a well-studied matrix previously developed at TU Dresden was used [16]. The mixture contains Portland cement CEM I 42.5 R-HS, 95% of pure clinker minerals, and fly ash steament H-4 as the binder material, quartz sand, water, a polycarboxyl ether-based superplasticizer MasterGlenium ACE 30 from BASF, and a viscosity-modifying agent, UW Compound-100 from SIKA, in order to achieve proper workability. The composition is given in Table 1.

The microfibre under investigation was the PVA fibre Kuralon K-II REC 15 from Kuraray, Japan with a nominal diameter of 40 µm. Further properties of the fibre are given in Table 2. Beside polyvinyl alcohol fibres, polyethylene (PE) and polybenzoxazole (PBO) fibres were also used for additional investigations.

### 2.2. Experiments

**Figure 1:**
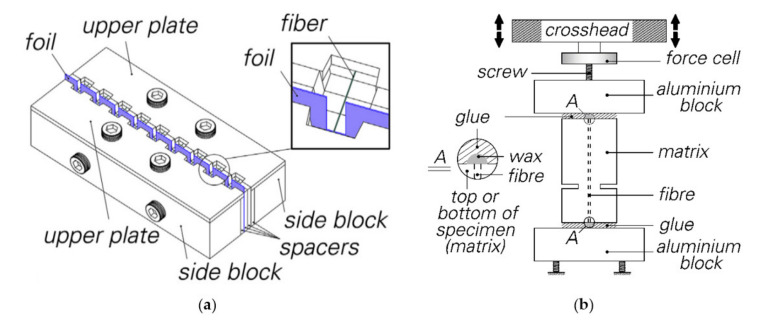
Double-sided, single-fibre pull-out [22] (Copyright permission are granted by the corresponding journal): (**a**) casting of the specimen; (**b**) testing setup.

To investigate the mechanical behaviour of the material on the single-fibre scale, especially under reversed cyclic loading, Ranjbarian and Mechtcherine [22] designed a new double-sided, single-fibre pull-out testing setup. Compared to the previous single-sided single fibre pull-out experiments, this setup enables the analysis of the degradation processes of fibres and matrix under an alternating tension-compression loading regime. In doing this, notched specimens were produced with a special mould consisting of several parts; see Figure 1a.

After casting, the specimens were stored in their moulds for not only one but two days, since the small samples are very sensitive and tend to break otherwise. After demoulding, the specimens were stored for a further 26 days under controlled climate conditions (20 °C, 65% RH) prior to testing. For testing, the specimens were fixed to the testing machine with a special, fast-setting glue, X60 from HBM, Darmstadt, Germany. In preparation for testing, the specimens were provided with a small drop of wax to prevent contact between fibre and glue. Figure 1b shows the complete test setup.

**Figure 2:**
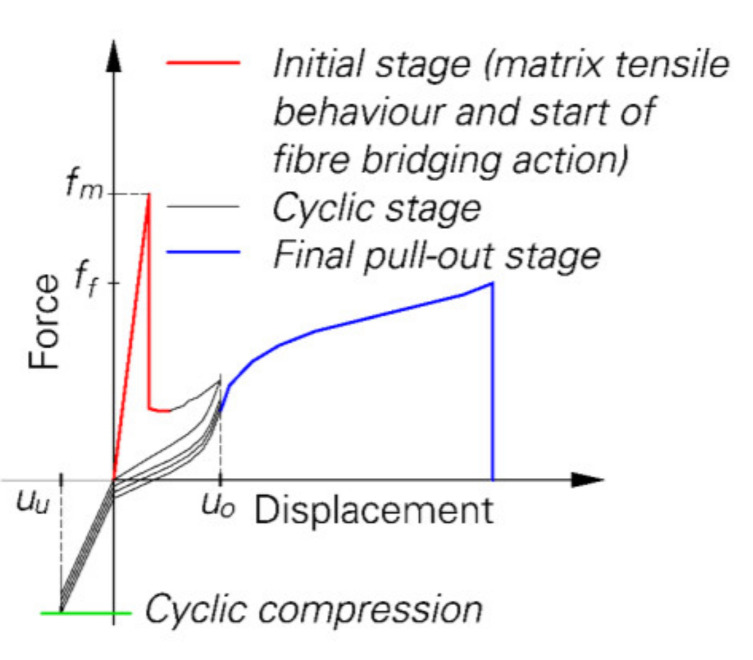
Loading regime for alternating tension-compression loading [23] (Copyright permission are granted by the corresponding journal).

To determine the cyclic mechanical behaviour of the material, a three-stage loading regime was applied to the specimens [23,24,29]; see also Figure 2. First, the sample was loaded under a quasi-static tension regime at a displacement rate of 0.01 mm/s until a crack opening of 100 µm was reached. Subsequently, the cyclic stage with a displacement rate of 1 mm/s started. At this stage, 0.1 mm was defined as the upper reversal point while 0.02 mm, −0.01 mm or −0.025 mm [23] or −10 µm [24] were set as lower reversal points in order to obtain different levels of compressive stress between the crack faces. After a predefined number of loading cycles, the fibre was pulled out at a displacement rate of 0.01 mm/s.

Ranjbarian and Mechtcherine [26] applied the same loading regime with distinct upper reversal points, e.g., 40 µm and 100 µm, to investigate the impact of the initial crack width on the performance of the embedded single fibre. In addition, the authors applied an incremental displacement per cycle (0.1 µm, 0.02 µm and 0.01 µm) depending on the number of loading cycles.

A further study on the cyclic behaviour of different polymer fibres by Ranjbarian and Mechtcherine [25] was performed using the same loading regime under a variation of the deformation-controlled upper and force-controlled lower reversal points, i.e., incremental displacement of 0.1 mm per cycle and compressive force of −15 N. After 500 loading cycles, the fibres were pulled out from the cement-based matrix.

One-sided, single-fibre pull-out tests were performed by Ranjbarian et al. [27] to assess the influence of the fibre inclination angle on the fibres’ behaviour under quasi-static and cyclic tension loading. The embedded and free lengths were set to 2 mm each. The small matrix block was fixed to an aluminium block attached to the testing machine’s crosshead with a predefined inclination angle of 0°, 30° or 60°. The free length of the fibre was clamped directly to the testing machine. The displacement rate for the quasi-static tests was 0.01 mm/s while the displacement rate for cyclic tests was 0.05 mm/s. The lower and upper reversal points for the tests were defined as 0.2 N and 0.5 N.

To investigate the pull-out behaviour of the PVA fibres after full debonding, Ranjbarian et al. [28] also performed single-sided pull-out tests until certain force levels were reached. An initial testing group was loaded to 55% of the force corresponding to full debonding. In other test series, the pull-out force reached a level of 70% or 95%, related to the force at rupture. When the defined force was reached, the matrix blocks were split by a foil inserted to gain access to the partially pulled-out fibre.

The mechanical experiments were supported by microscopic investigations performed with an environmental scanning electron microscope Philips XL 30.

### 2.3. Numerical Methods

Material degradation of SHCC on the micro-scale is mainly driven by fracture and damage processes in the concrete matrix, by fibre debonding and pull-out, and by fibre damage mechanisms as well. Different degradation mechanisms are activated for quasi-static and cyclic loading and under different loading regimes. Hence, micromechanical models must describe the characteristic deformation processes of concrete, fibre and concrete–fibre interface.

**Figure 3:**
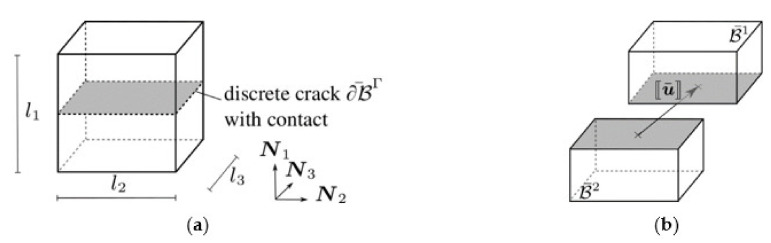
Representative crack element in: (**a**) undeformed and (**b**) deformed configuration.

Nucleation and propagation of cracks and crack networks in high- and ultra-high-strength concrete are modelled using various methods in the literature, e.g., the particle-bond method, meshfree methods such as the optimal-transportation method or the material point method, and the finite element method (FEM). The last approach is applied to cohesive zone models, nonlocal damage models, eXtended Finite Element Method (XFEM), regularised formulations of the free-discontinuity problems like phase-field fracture and eigenfracture. A phase-field formulation for fatigue crack propagation under cyclic loading was introduced by Carrara et al. [30] and the simulation is accelerated by means of the cycle jump method in Schreiber et al. [31] and Loew et al. [32]. The phase-field method for fracture is used in what follows to describe the fracture processes in the concrete matrix. In comparison to the other above-mentioned models, fewer assumptions in the formulation are necessary to describe crack nucleation and crack propagation under static and cyclic loads. The phase-field model is shown to represent the energetic description of discrete cracks introduced by Griffith at the limit where the regularisation length approaches zero [33,34]. However, a small regularisation length can only be realised on a fine mesh in the environment of cracks and locations where cracks nucleate. Although the numerical solution procedure has been significantly accelerated in some recent publications [35,36], the convergence behaviour is comparably slow. The phase-field fracture formulation in the form

(1) $$E=\underset{B}{\int }\left[\psi^{c}+g\left(p\right)\;\left(\psi^{0}-\psi^{c}\right)\right]dV+G_{c}\underset{B}{\int }\gamma \left(p\right)\;dV$$

is used in the following numerical simulations. The integral in the last term is the regularised crack surface area. The first term defines the deformation energy as the linear super-position of $\psi^{0}$ and $\psi^{c}$ based on the degradation function $g\left(p\right)$. The two energies $\psi^{0}$ and $\psi^{c}$ are related to the material states with and without a crack, i.e., $\psi^{0}$ is an ordinary constitutive model and $\psi^{c}$ describes the material behaviour in the presence of a crack. The decomposition of the deformation energy is performed in most phase-field models by means of artificial split approaches, commonly by a decomposition of the strain or stress tensor. Those splits are shown to be imprecise or even to yield unphysical predictions. Alternatively, the cracked material behaviour is derived from a representative model of a small portion of the crack; compare Figure 3. The concept of representative crack elements (RCE) is introduced in Storm et al. [37] and has proven to be reliable and very flexible [38,39,40]. 

The fibre model is first applied to quasi-static pull-out tests. Transversal-isotropic hyper-viscoelasticity at finite deformations is considered for the constitutive model

(2) $$\psi \left(C\right)=\overset{n}{\underset{i=2}{\sum }}a_{i}{\left(I_{4}\right)}^{i},$$

where the deformation invariant $I_{4}$ is related to the right Cauchy–Green tensor C and the fibre direction a by $I_{4}=a\cdot C\cdot a$. The generalised Maxwell model

(3) $$H_{j}^{n+1}=exp\left(-\frac{\Delta t}{\tau_{j}}\right)H_{j}^{n}+\gamma_{j}\frac{1-exp\left(-\frac{\Delta t}{\tau_{j}}\right)}{\frac{\Delta t}{\tau_{j}}}\left(dev\;S_{0}^{n+1}-dev\;S_{0}^{n}\right)$$

(4) $$dev\;S^{n+1}=dev\;S^{n}+\overset{N}{\underset{j=1}{\sum }}H_{j}^{n+1}$$

is adopted for the time-dependent deformation behaviour; compare Kaliske and Rothert [41]. Specific damage processes like fibre fatigue, fibre abrasion, fibre buckling and defibrillation and fibre squeezing are relevant at cyclic loading and will be considered in ongoing research. A major challenge in SHCC is the high number of fibres in the composite. On the one hand, the high number of fibres opens the possibility of homogenised macroscopic models where single fibres are not resolved explicitly. On the other hand, most inelastic deformation processes take place at the concrete–fibre interface, in the fibre and in the neighbourhood of the fibre.

**Figure 4:**
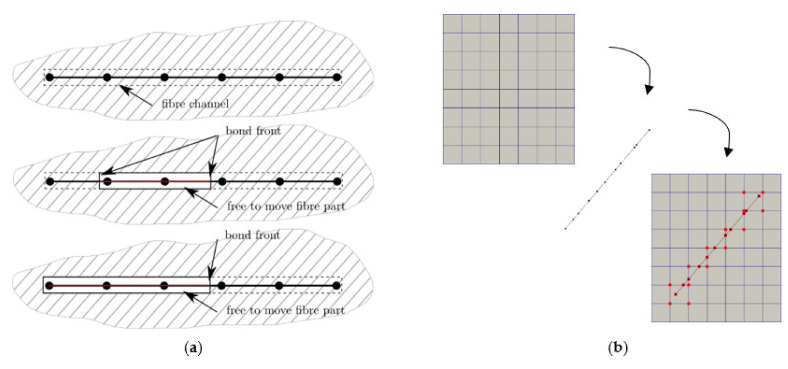
(**a**) Considered fibre bond states at the fibre super-element, (**b**) coupling of concrete mesh and fibre super-element by means of kinematic constraints.

Therefore, single fibres have to be modelled in micromechanical studies. Reliable predictions based on micromechanics can be achieved, but computational costs increase rapidly with the number of fibres in the model when the fibres are modelled by solid elements and the concrete–fibre interfaces by interface elements. Alternatively, fibres can be modelled by means of structural formulations such as beam elements, and the coupling to the concrete by displacement-slip models. In this way the computational costs are reduced by at least one order of magnitude. However, this approximation barely represents the degradation processes described at the concrete–fibre interface. Moderate computational effort is also achieved when the fibre and the concrete–fibre interface are modelled by a specialised element formulation, called the fibre super-element; compare Storm et al. [42]. The super-elements are coupled to the concrete mesh through kinematic constraints, which avoid excessive refinement of the concrete mesh towards the fibres; see Figure 4.

## 3. Results and Discussion

**Figure 5:**
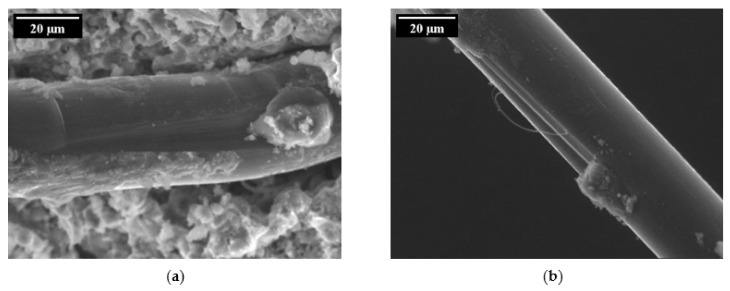
Damage types on the surface of a PVA fibre [28] (Copyright permission are granted by the corresponding journal): (**a**) micro-excavation and bump; (**b**) separated fibrils.

Single-sided, single-fibre pull-out tests under a quasi-static loading regime revealed a significant degradation process of the PVA fibre during pull-out from a cement-based matrix. Ranjbarian et al. [28] found two damage types resulting from pull-out. The first is the so-called “micro-excavation and bump” type; see Figure 5a. Here, parts of the fibre’s skin are peeled off and accumulate on the surface behind this area. The second observation notes “separated fibrils” caused by partial abrasion during pull-out process; see Figure 5b. By analysing the observations of the mechanical tests at different force levels, the authors were able to explain the initiation process of the occurring damages. After full debonding of the fibre, however, there are still spalled matrix particles adhering to the fibre’s surface caused by the strong chemical bond between PVA fibre and matrix. When pulling the fibre out of the cement-based matrix, these particles are pressed into the fibre, causing local scratching. The fibre material removed accumulates behind this area, initiating the development of a bump. Due to the development of this bump, the fibre is partially locked in the fibre tunnel, thus preventing further fibre pull-out without further fibre degradation. The micro-excavation leads to the reduction of the cross-section and may therefore promote the rupture of the PVA fibre in this region.

**Figure 6:**
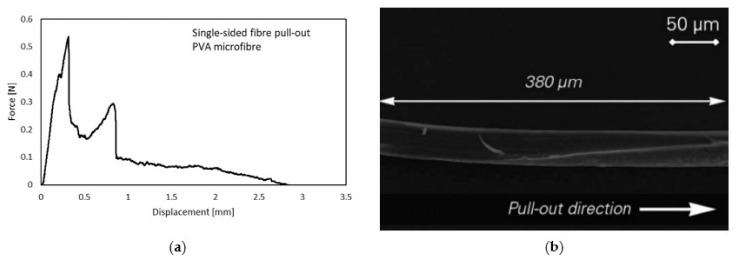
Results of single-sided, single-fibre pull-out test with complete fibre pull-out instead of fibre rupture [28] (Copyright permission are granted by the corresponding journal): (**a**) mechanical behaviour; (**b**) long micro-excavation on the embedded part of the PVA fibre.

Regarding the results of the single-fibre pull-out tests, slip-softening behaviour could also be observed instead of an abrupt drop in force, indicating fibre pull-out rather than fibre rupture after a slip-hardening stage; see Figure 6a. In explaining this behaviour, a numerical study based on a cohesive interface model and microscopic analyses of the fibre surface was performed [28]. Interestingly, the authors found a large micro-excavation (380 µm in length) without the previously described bump of accumulated fibre material extending to the end of the embedded part of the fibre; see Figure 6b. A possible explanation by Ranjbarian et al. [28] is the shearing off of the bump near the end of the fibre, resulting in an abrupt drop in force and the subsequent pull-out of the fibre from the matrix.

The pull-out mechanisms identified are considered for the super-element formulation. Nucleation and propagation of de-bonded fibre regions in the fibre channel are explicitly modelled and the deformation of the debonded fibre parts depend on friction, locking points and fibre-end anchoring.

**Figure 7:**
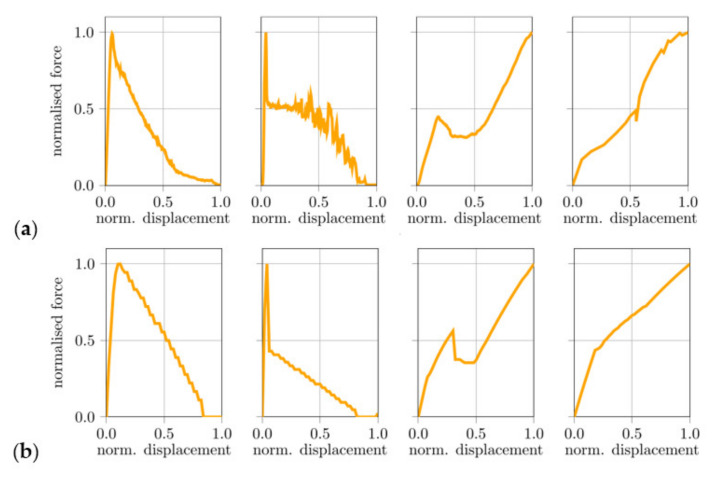
Force-displacement relations for the cases of total fibre pull-out (Type 1), total pull-out (Type 2), pull-out with fibre locking and pull-out with fibre-end anchorage (from left to right) obtained from (**a**) pull-out experiment with PE and PVA fibres and (**b**) numerical simulation with the fibre super-element.

Characteristic pull-out curves obtained from pull-out tests with PE and PVA fibres are shown in Figure 7 and compared to corresponding numerical results using the fibre super-element.

**Figure 8:**
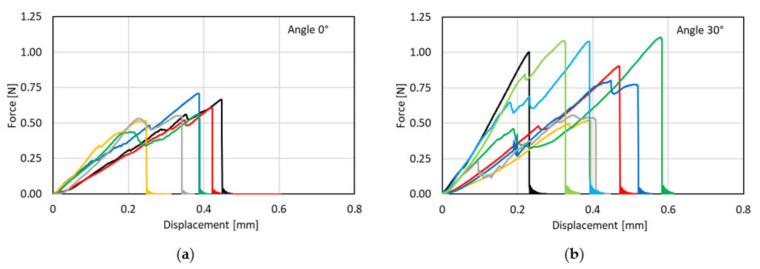
Pull-out force-displacement curves for different inclination angles [27] (Copyright permission are granted by the corresponding journal): (**a**) 0°; (**b**) 30°.

Ranjbarian et al. [27] observed the dependence of the mechanical performance of PVA microfibres on their angle of inclination in single-sided fibre pull-out tests. Compared to non-inclined fibres, fibres with a higher inclination angle show more pronounced scattering in the force-displacement curves, with multiple drops probably caused by the progressive damage in the transition area between free fibre length and embedded fibre length; see Figure 8.

By analysing the test results, the authors also concluded an increase in force at rupture with increasing fibre inclination angle. Moreover, slight increases in displacement-at-fibre-rupture and pull-out-work could be observed. Considering the ESEM-images of the ruptured sections, the authors concluded flexural failure of the fibre in the vicinity of the interface between free and embedded lengths of fibre with an inclination angle of 30° or 60°. Here, material from the fibre surface peeled off during pull-out and accumulated nearby.

**Figure 9:**
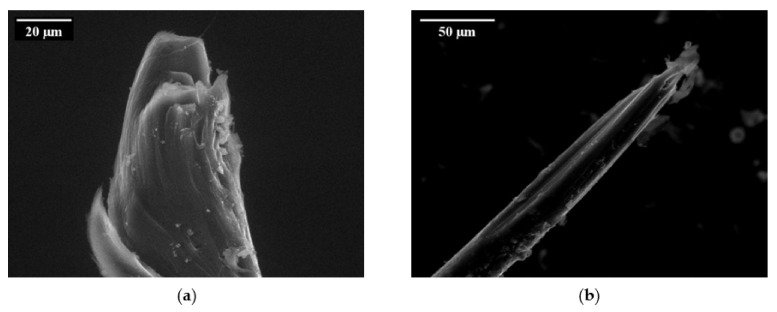
Ruptured sections of fibres with different inclination angles [27] (Copyright permission are granted by the corresponding journal): (**a**) 60°; (**b**) 0°.

**Figure 10:**
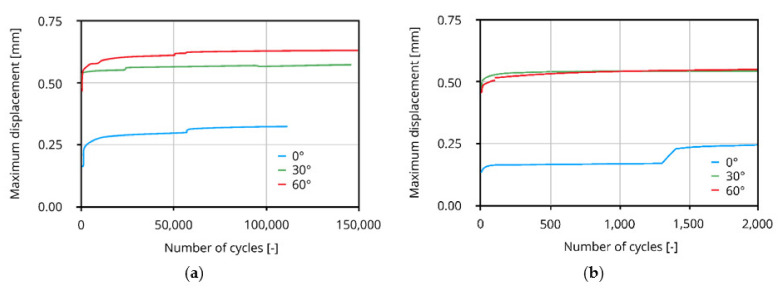
Modified schematic maximum displacement curves over number of cycles modified after [27] (Copyright permission are granted by the corresponding journal): (**a**) complete cyclic loading stage; (**b**) early loading cycles.

Due to stress concentrations at the deflection points and reduced cross-sections, the fibres are likely to rupture; see Figure 9a. In contrast, the failure of straight fibres follows the above-described locking front model with long abrasion stripes and fibre failure located in the fibre tunnel; see Figure 9b. Figure 10 illustrates the maximum displacement versus the number of loading cycles in a pure cyclic tension regime.

As under quasi-static loading, the maximum displacement increases with an increasing fibre inclination angle. All three test-series show similar behaviour with regard to a higher increase in displacement in earlier cycles and a less pronounced increase in displacement in later loading cycles. Considering the non-inclined PVA microfibres, a sudden increase in displacement can be observed in the early cycles in contrast to the inclined fibres caused by full debonding of the fibres from the cementitious matrix; see Figure 10b.

Nevertheless, the authors could not identify any significant influence of fibre inclination angle on cyclic behaviour.

After investigating the pull-out mechanisms and degradation processes of PVA fibres by single-sided single-fibre pull-out tests in quasi-static and pure cyclic tension regimes, Ranjbarian and Mechtcherine [23,24,25,29] performed double-sided fibre pull-out tests in monotonic and cyclic tension-compression regimes.

**Figure 11:**
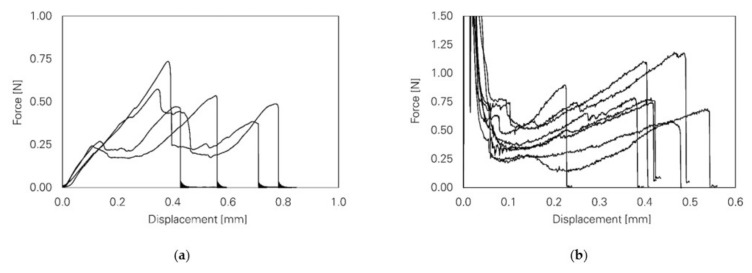
Single fibre pull-out response [22] (Copyright permission are granted by the corresponding journal): (**a**) single-sided pull-out test; (**b**) double-sided pull-out test.

To verify the newly designed double-sided, single-fibre pull-out test setup, Ranjbarian and Mechtcherine [22] carried out single-sided and double-sided pull-out tests with an embedded length of 2 mm as well as single-fibre tension tests. The results of the pull-out tests are shown in Figure 11.

**Figure 12:**
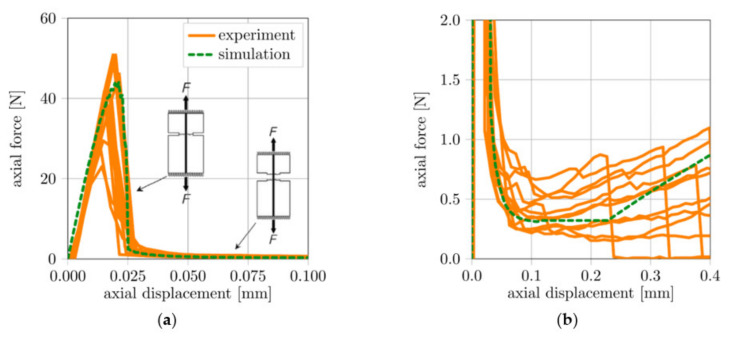
Force-displacement characteristic of the numerical simulation of the doubled-sided pull-out test, (**a**) reaction force by concrete fracture and (**b**) fibre pull-out beyond concrete fracture.

Considering Figure 11b, after failure of the cementitious matrix, the same behaviour as with single-sided fibre pull-out occurs; i.e., the force drops due to the loss of the chemical bond between fibre and matrix, before the fibres rupture. Hence, the authors concluded that the new setup delivers reliable results. Additionally, it could be shown that the double-sided, single-fibre pull-out behaviour enables the investigation of the mechanical behaviour and degradation mechanisms under alternating cyclic loading. The double-sided pull-out test was numerically studied, applying the phase-field method for concrete fracture and the super-element for fibre deformation and fibre pull-out; compare Storm et al. [42] and Figure 12.

In a preliminary study, Ranjbarian and Mechtcherine [24] investigated the cyclic damage to PVA-fibres caused by alternating cyclic loading. Lengths of 100 µm and −10 µm were chosen as upper and lower reversal points for tests with a constant displacement rate of 0.01 mm/s. After reaching a pre-defined number of cycles, i.e., 200 and 2000 (C200 and C2000), the fibre was finally extracted.

**Figure 13:**
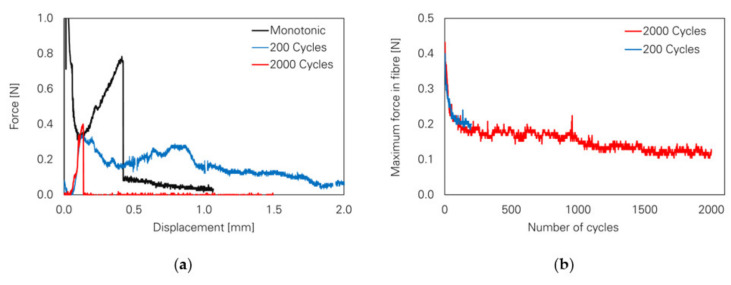
Mechanical behaviour of the PVA fibres under cyclic loading [24] (Copyright permission are granted by the corresponding journal): (**a**) fibre pull-out behaviour caused by reversed cyclic loading; (**b**) maximum force in the fibres over the entire load stage.

**Figure 14:**
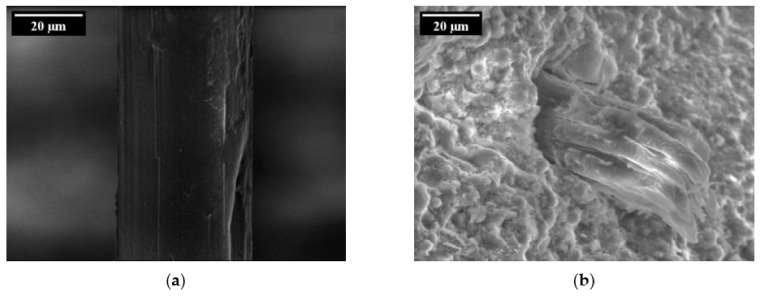
Damage types on PVA fibres under cyclic loading [24] (Copyright permission are granted by the corresponding journal): (**a**) delamination and abrasion; (**b**) fibre rupture due to severe defibrillation and buckling of the fibre between the crack faces.

By changing the loading regime from quasi-static to alternating cyclic loading, a different pull-out behaviour could be observed in the case of the test series loaded with 200 cycles; see Figure 13a. Here, the fibre is pulled out of the cement-based matrix instead of rupturing, as occurs in the case of monotonic loading with significant probability. Nonetheless, an increase in the number of cycles up to 2000 also causes rupture of the PVA fibre subject to significantly lower forces and maximum displacements compared to fibres pulled out in a quasi-static loading regime; see Figure 13b. Furthermore, a decreasing trend in the maximum force in the fibres can be observed with an increasing number of cycles due to relaxation phenomena in the fibre–matrix interface or the fibre itself. The microscopic analysis of the specimens revealed specific damage to the fibres after alternating cyclic loading. In the case of completely extracted fibres (C200), they showed signs of delamination and abrasion located at the interface of the embedded part and the crack face, whereas defibrillation could be observed between the crack faces; see Figure 14a. Due to repeated opening and closing of the crack in the reversed cyclic loading, the fibres were squashed and buckled between the crack faces, creating a weak zone by increasing defibrillation and abrasion. Hence, in the final pull-out stage the fibre ruptured; see Figure 14b.

**Figure 15:**
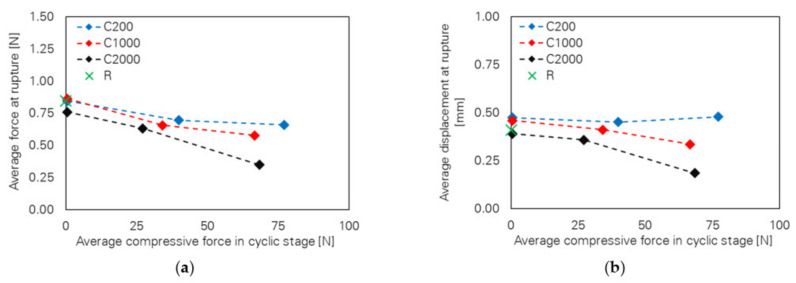
Effect of compressive stress level and number of loading cycles [43] (Copyright permission are granted by the corresponding journal): (**a**) force at rupture; (**b**) displacement at rupture.

A further study conducted by the authors of [23] puts its focus on the cyclic response of a single PVA fibre under different numbers of loading cycles and variation of the lower reversal points, i.e., 0.020, −0.010 and −0.025 mm. In addition to the previous study, the evaluation of the results showed the occurrence of fibre rupture with one-way and two-way full debonding, i.e., the fibre was debonded from both embedded parts. Aside from that, complete fibre pull-out could be observed for a few samples. Considering the average force at rupture as well as the average displacement at rupture depending on the average compression during the cyclic loading stage, clear trends could be identified; see Figure 15. An increase in compressive force led to a decrease in the force at rupture and displacement at rupture that became more significant with a simultaneous increase in the number of loading cycles. 

**Figure 16:**
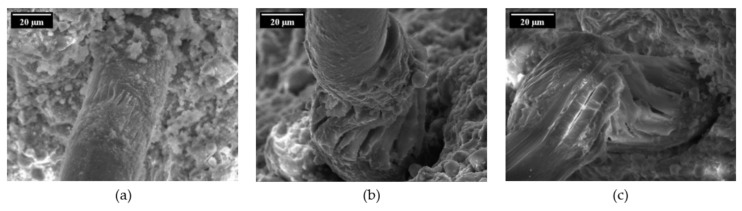
Damage types of the fibre subject to reversed cyclic loading with different compressive stress levels [23] (Copyright permission are granted by the corresponding journal): (**a**) longitudinal cracks in the fibre and abrasion in pure cyclic tension regime after 2000 cycles; (**b**) severe fibre buckling and defibrillation at moderate compressive stress level (−0.01 mm) after 2000 cycles; (**c**) severe fibre buckling and defibrillation at high compressive stress level (−0.025 mm) after 1000 cycles.

Such behaviour can be explained by different degradation stages observed by means of a scanning electron microscope. As stated above, the cyclic loading leads to a shift of the damage mechanism detected in quasi-static pull-out tests. In the case of purely cyclic tension tests, the fibres show small longitudinal cracks due to lateral extension and local compressive stresses caused by local deviations from the global stress state and friction between the fibres and the fibre tunnel near the crack face; cf. Figure 16a. By increasing the number of loading cycles, these longitudinal cracks lead to the defibrillation of the PVA fibres. When applying an alternating cyclic loading regime with moderate compression, additional damage mechanisms such as buckling or superficial abrasion contribute to the deterioration of the fibres, thus causing a higher extent of damage when compared to purely cyclic tension regimes; see Figure 16b. A high cyclic compressive force induces the severe fibre damage already described. Several damage types could be observed after cyclic loading of the specimens such as pronounced defibrillation, buckling and superficial signs of deterioration caused by friction especially between the crack faces; see Figure 16c.

By analysing the results from the alternating cyclic double-sided, single-fibre pull-out tests, the authors derived damage indices for pull-out toughness, pull-out force and displacement at rupture, i.e., δ_p_, δ_r_ and δ_d_. These cyclic damage indices were examined by curve-fitting depending on the number of loading cycles N and the compressive stress level σ_c_. The indices thus determined enabled the prediction of the bearable number of cycles at certain compressive stresses.

Since the study suggests that crack width has a significant influence on the buckling and defibrillation of the fibre and, therefore, on fibre failure, Ranjbarian and Mechtcherine [26] investigated the influence of the initial crack width on the fibre degradation with the same test setup as before. The authors recognised a clear dependence of fibre degradation on crack width. The results showed that mechanical indices differed for small (40 µm) and large (100 µm) crack widths, i.e., pull-out toughness, force at rupture and displacement at rupture.

**Figure 17:**
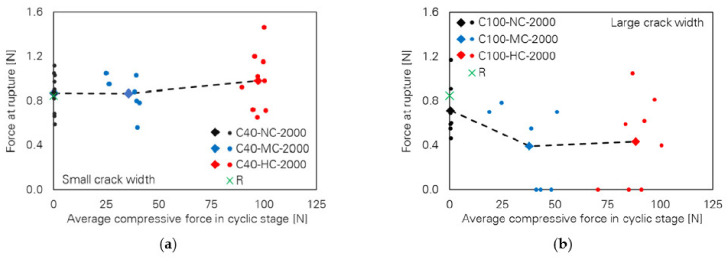
Influence of crack width on the force at rupture of PVA fibres subjected to 2000 loading cycles depending on lower reversal point [26] (Copyright permission are granted by the corresponding journal): (**a**) small crack width (40 µm); (**b**) large crack width (100 µm).

**Figure 18:**
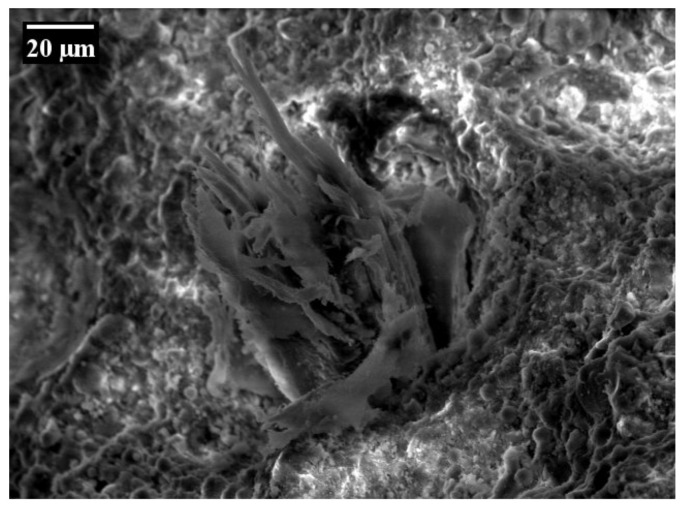
Ruptured section of the PVA fibre tested with a large crack width [26] (Copyright permission are granted by the corresponding journal).

Figure 17 shows the force at rupture of the PVA fibres for different crack widths depending on the number of cycles and on the lower reversal point. Considering the results from the cyclic pull-out tests with small initial crack widths (Figure 17a), only a minor impact of the compressive stress levels can be seen on the force at rupture. Perhaps this crack width is too small to induce full debonding of the fibres from the cementitious matrix. Hence, the fibre would just deform elastically at the tensile-load stage and could simply deform back when the specimen is compressed. Even if full debonding were to occur, the crack width would not be sufficient to initiate defibrillation of the fibre. Thus, the impact of the compressive stress is negligible. In contrast, fibre deterioration is more severe for large crack widths. As can be seen in Figure 17b, force at rupture generally decreases with increasing compressive force for a large initial crack width. It can be assumed that the fibre is already fully de-bonded and has slipped out of the tunnel before the cyclic stage begins. When the sample is compressed, the fibre cannot be pushed back into the fibre tunnel. Therefore, the fibre is bent in the compression stage and is finally crushed between the crack faces. Due to this fibre degradation, fibres tend to rupture already in the cyclic stage when the initial crack width is large. Since the PVA fibres possess poor mechanical properties in the transverse direction, a “fibrillar” fracture type can be observed [26]; see Figure 18.

Samples tested at small crack widths show a higher force at rupture since only slight deterioration occurs, as the fibre can simply deform back into the fibre tunnel in the compressive loading stage. The force at rupture is significantly smaller for large crack widths since the fibres suffer from severe damage caused by repeated crushing and bending of the fibre between the crack faces. Due to the more severe fibre degradation, the fibres’ mechanical behaviour is strongly affected. Hence, the results from the cyclic tests with large initial crack widths show more pronounced scattering.

Due to the significant influence of the initial crack width on mechanical behaviour and fibre deterioration, the damage quantification approach derived in [23] mentioned above had to be extended to include another variable, i.e., crack width (ω). Detailed information about the algorithm can be found elsewhere [26].

**Figure 19:**
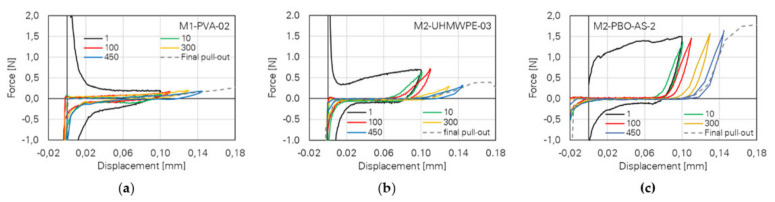
Representative results of cyclic pull-out tests with different fibres [25] (Copyright permission are granted by the corresponding journal): (**a**) PVA; (**b**) UHMWPE; (**c**) PBO–AS.

In addition to investigations of the cyclic behaviour of PVA fibres, Ranjbarian and Mechtcherine [25] performed cyclic multiple fibre pull-out tests on ultra-high-molecular-weight polyethylene (UHMWPE) and poly(p-phenylene-2,6-benzobisoxazole) (PBO-AS) fibres. The tests showed different crack-bridging behaviours of the polymer fibres. The corresponding results are displayed in Figure 19.

While PVA fibres show relatively low pull-out forces, higher force levels can be observed for UHMWPE fibres caused by the high-strength concrete matrix used for UHMWPE and PBO–AS fibres due to their hydrophobic properties. Here, a decrease in pull-out force can be recognized in later loading cycles due to the rupture of individual fibres. PBO–AS fibres show the highest maximum pull-out forces with relatively constant values in the depicted loading cycles; see Figure 19c. Subsequently, the samples were investigated using an ESEM. For the specimens with UHMWPE fibres, defibrillation and an increase in the diameter of the fibre could be recognized, whereas PBO fibres showed only slight signs of deterioration.

## 4. Conclusions

Strain-hardening cement-based composites (SHCC) are a promising class of construction materials for practical applications, especially under dynamic and cyclic loading conditions. To ensure the safe use of the material, sound knowledge of the material’s behaviour and degradation processes on the material level is required.

For this reason, several studies have been conducted on the single-fibre scale of PVA–SHCC to gain findings about the damage mechanisms under quasi-static and cyclic loading depending on various parameters, i.e., fibre orientation, initial crack width or number of loading cycles. Fibre rupture was the main failure mode under quasi-static loading. Based on the microscopic analyses, the so-called locking-front model was developed, describing the interlocking of the fibre in the fibre channel due to the abrasion of fibre material and its accumulation between the fibre’s surface and the channel. By changing the loading regime from quasi-static to reversed cyclic loading, a shift in the degradation mechanisms could be observed. Instead of fibre rupture caused by the locking of the fibre in the fibre channel, multiple signs of deterioration could be observed in the cyclic loading regime. Due to the repeated opening and forceful closing of the crack—especially with large initial crack widths—the fibre is buckled and crushed between the crack faces, thus causing progressive defibrillation and final rupture of the fibre.

Additionally, a micromechanical model based on the phase-field approach with representative crack elements (RCE) was implemented to support the understanding of the experimental findings. By introducing the fibre super-element, the computational effort could be significantly reduced.

The experiments conducted and the related findings on the degradation processes in SHCC on the micro-scale contribute to a better understanding of the material’s behaviour under cyclic loading. Analytical descriptions and extensive numerical simulation enable the prediction of the composite’s behaviour under various loading states and allow the optimisation of the material.

## Figures and Tables

**Table 1:** Composition of the normal-strength SHCC matrix, in kg/m^3^.

Cement CEM I 42.5 R-HS	Fly Ash	Water	Quartz Sand 0.06/0.2	Superplasticizer	Viscosity Agent
505	621	338	536	10	4.8

**Table 2:** Salient properties of PVA fibre.

Nominal Diameter [µm]	Tensile Strength [MPa]	Young’s Modulus [GPa]	Strain Capacity [-]	Density [g/cm^3^]
40	1600	40	0.07	1.3
